# A variance component-based gene burden test

**DOI:** 10.1186/1753-6561-8-S1-S49

**Published:** 2014-06-17

**Authors:** Juan M Peralta, Marcio Almeida, Jack W Kent, John Blangero

**Affiliations:** 1 Department of Genetics, Texas Biomedical Research Institute, 7620 NW Loop 410, San Antonio, Texas 78227-5301, USA; 2 Centre for Genetic Origins of Health and Disease of Western Australia (M409), 35 Stirling Highway, Crawley, WA 6009, Australia

## Abstract

We propose a novel variance component approach for the analysis of next-generation sequencing data. Our method is based on the detection of the proportion of the trait phenotypic variance that can be explained by the introduction of a new variance component that accounts for the local gene-specific departure of the empirical kinship relationship matrix, estimated from single-nucleotide polymorphism (SNP) genotypes, from their theoretical expectation based on the genealogical information in the pedigree. We tested our method with simulated phenotypes and imputed SNP genotypes from the Genetic Analysis Workshop 18 data set. We observed considerable variation in the differences between theoretical and gene-specific kinship estimates that proved to be informative for our test and allowed us to detect the *MAP4 *causal gene at a genome-wide significance level. The distribution of our test statistic show no inflation under the null hypothesis and results from a random set of genes suggest that the detection of MAP4 is both sensitive and specific. The use of 2 different strategies for the selection of the SNPs used to derive the gene-specific empirical kinship relationship matrices provides us with suggestive evidence that our method is performing as an empirical test of linkage.

## Background

Complex phenotypes are thought to be determined by the aggregate effects of many rare causal variations [1-3]. Detection of the true causal variations present in next-generation sequencing data sets [4,5] is challenging because their faint signals are difficult to separate from background noise. Most of the current analytical methods try to improve the signal-to-noise ratio by reducing the number of statistical tests needed for a significant signal to be detected.

A common approach to alleviate the multiple-testing problem is to collapse, commonly by membership of a variant in a known annotated gene or pathway, the information conveyed by individual variants into a single measure, like a principal component or a weighted rank, that can then be tested [6]. However, a common limitation of many approaches is that the aggregation of the variants into a single measure often involves an arbitrary definition of the directionality of each variant's fixed effects.

We present a novel random-effect-variance component-based approach that uses gene-specific relationship matrices to collapse variants into a per-gene genetic contribution effect.

## Methods

### Data set

The Genetic Analysis Workshop 18 (GAW18) data [7], based on whole genome sequencing data for the odd-numbered chromosomes of 464 individuals released by the T2D-GENES Consortium, was used to test our method. Specifically, we used pedigrees, minor allele-based single-nucleotide polymorphism (SNP) dosages, and the SIMPHEN.1 simulated phenotypes in the GAW18 data set.

### Definition of the gene loci

The transcription start site and the stop codon coordinates for the longest transcript associated with a gene were obtained from the UCSC's human genome release 19 (hg19) known gene table.

### Gene-specific SNP dosages

To investigate if the procedure used to select the SNPs that were collected on a per-gene locus basis affected our test results, we used 2 different SNP selection approaches: the intragenic and the nonsyn strategies. The intragenic strategy consisted of the selection of all SNPs within the bounds of a gene. The nonsyn strategy consisted of the selection of the subset of intragenic SNPs that were annotated as being nonsynonymous coding changes using ANNOVAR [8]. GAW18 SNP dosages from the imputed genotypes where then collected into separate, gene-specific, dosage files for SNPs selected using the intragenic and nonsyn strategies.

### Gene-specific empirical kinship matrices

Gene-specific dosages were transformed into genotypes and processed with KING [9], a method for relationship inference from large SNP genotype data sets that is robust to population substructure, to produce a gene-specific matrix of empirical kinship coefficients.

### Control for unknown population substructure

To control for possible population stratification, principal component loadings were calculated using the prcomp function in R [10], with data from 117 unrelated individuals for approximately 29,000 haplotype tagging SNPs in low mutual linkage disequilibrium, and then projected onto the full set of genotyped individuals. The first 5 principal components explained 5% of the total phenotypic variance and were added as covariates to our variance component model.

### Trait and covariates

We used the simulated phenotypic data at the first exam for the systolic blood pressure (SBP_1) trait. The sex (SEX), age (AGE_1), and smoke (SMOKE_1) status at the first exam phenotypes were introduced as covariates into our variance component model. The Q1 trait was used to assess the distribution of our test statistic under the null hypothesis.

### Variance component model

Our method uses gene-specific relationship matrices (GSRMs) to extract the proportion of the trait's variance explained by a single gene as a result of the departure of its localized empirical kinship estimates (EKEs) from their pedigree-derived theoretical kinship expectations (TKEs). A new variance component parameter ($h_{geff}^{2}$) was introduced into a standard variance component model

$$\Omega =\sigma_{Phenotypic}^{2}\left(2\Phi h_{r}^{2}+2Eh_{geff}^{2}+Ie^{2}\right)$$

where Ω is the covariance matrix, $\sigma_{Phenotypic}^{2}$ is the total phenotypic variance; $h_{r}^{2}$, $h_{geff}^{2}$, and $e^{2}$, respectively, represent the proportion of $\sigma_{Phenotypic}^{2}$ that can be attributed to the residual additive effect of polygenes, a gene-specific effect; and a random environmental effect, Φ, is the TKE kinship matrix, *E *is the EKE kinship matrix, and *I *is the identity matrix. This partitioning of the trait variance was estimated using an extension of the polygenic command from SOLAR [11] independently for each gene. The significance of each $h_{geff}^{2}$ estimate was obtained from a likelihood ratio test against the null model

$$\Omega =\sigma_{Phenotypic}^{2}\left(2\Phi h_{r}^{2}+Ie^{2}\right)$$

Because the variance component $h_{geff}^{2}$ is tested on its boundary, the likelihood ratio test statistic is distributed as a ½:½ mixture of a 1 degree of freedom (DF) chi-square and a point mass at zero [12].

## Results

**Figure 1:**
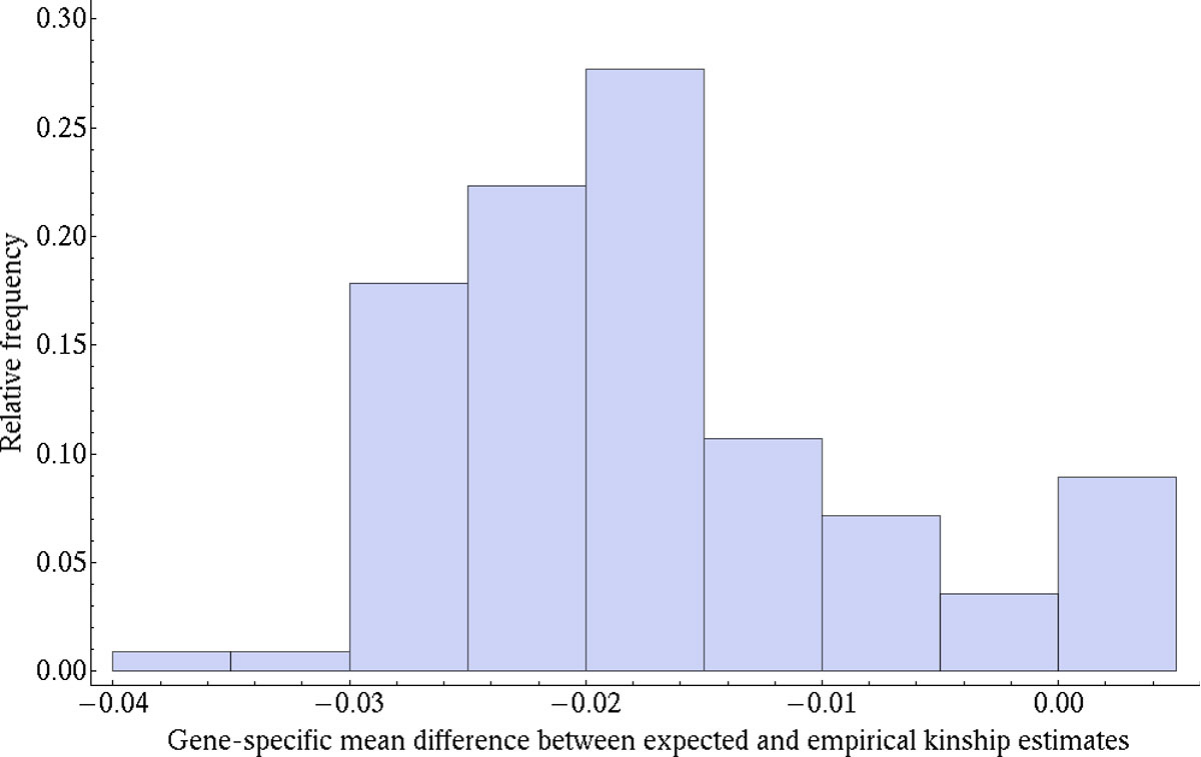
**Distribution of the gene-specific differences between TKEs) and EKEs**. Differences between TKE and EKE values were averaged by gene for a sample of 100 random and 12 SBP_1 causal genes. The negative sign indicates that the gene-specific EKE average is larger than the TKE average.

We compared the observed gene-specific EKE values obtained from the imputed SNP dosages with the TKE values derived from the pedigree and found substantial differences between them (Figure 1). The negative skew in Figure 1 shows that gene-specific EKE values are larger than their TKE counterparts and it shows that for certain genes individuals appear to be more closely related than expected from their relatedness in the pedigree.

**Figure 2:**
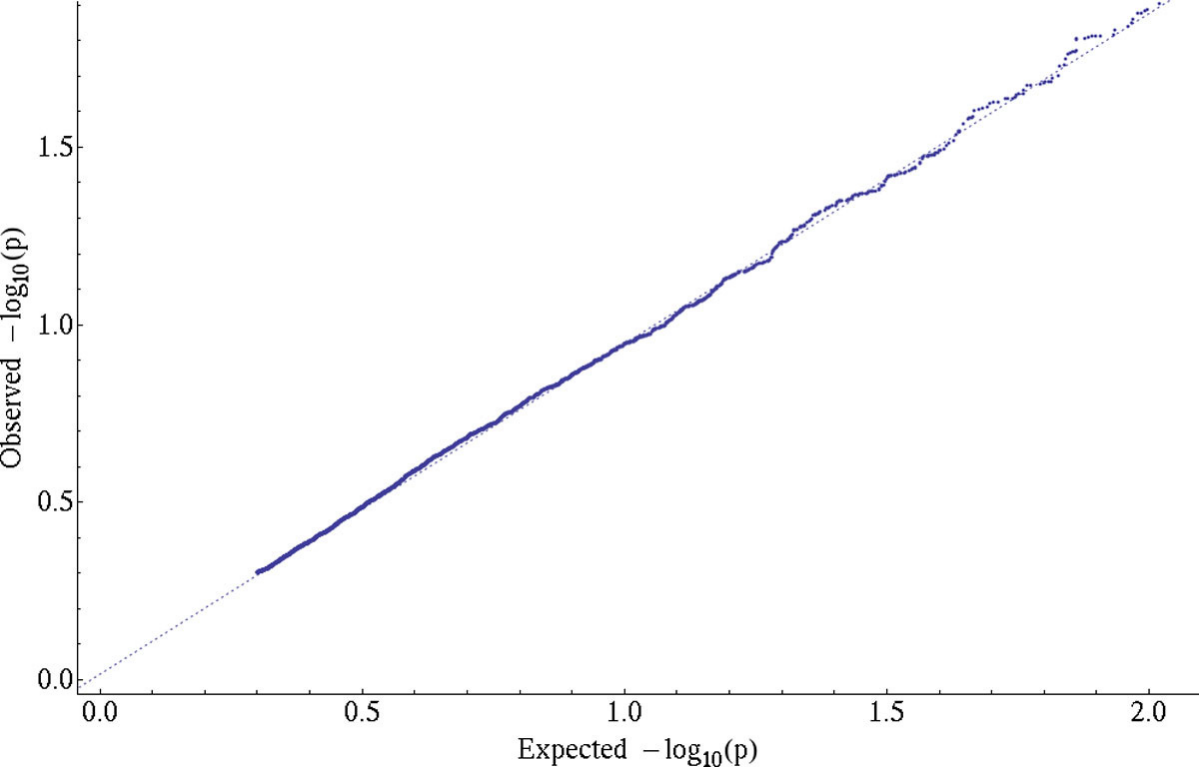
**Q-Q plot of the *p *values for the gene-specific effect estimates evaluated under the null hypothesis**. The *p *values for the gene-specific effect estimates were calculated using SNPs selected with the intragenic strategy for a random sample of 5000 genes, using the Q1 trait, a trait highly heritable but not influenced by any of the GAW18 SNPs.

We then performed variance component analyses using GSRMs with intragenic and nonsyn EKE values for 12 of the causal SBP_1 genes in the simulated data set (Table 1) and a random gene sample (Table 2). We detected a clear and significant signal from the *MAP4 *causal gene using both the intragenic and nonsyn strategies, that reached genome-wide significance (after a conservative Bonferroni correction for 30,000 tests, p <1.6 × 10^−6^) in the nonsyn (Table 1). The magnitude of the *MAP4 *signal is strong enough for it to be specifically detected as the top result in a random sample of 100 genes (Table 2). Other causal genes also rank among the top results, but their signals are weaker (Table 2). Figure 2 suggests that our approach has the sensitivity to separate true-positive signals from false-positive ones, as there is no inflation or deflation of the *p *values that we obtained for the estimates of the gene effects evaluated under the null hypothesis.

**Table 1:** Estimated effects on the simulated SBP_1 trait for known causal genes

Gene	Strategy							
	
	Intragenic				Nonsyn			
	
	h2r	h2r_p	geff	geff_p	h2r	h2r_p	geff	geff_p
*MAP4*	0.17	3.90 × 10^−6^	0.10955	7.20 × 10^−6^	0.18	7.00 × 10^−7^	0.10382	1.00 × 10^−7^
*LEPR*	0.26	4.16 × 10^−8^	0.04702	6.52 × 10^−3^	0.31	2.28 × 10^−10^	0.01147	1.71 × 10^−1^
*LRP8*	0.28	6.97 × 10^−9^	0.03575	6.55 × 10^−3^	0.32	3.44 × 10^−11^	0	1
*GTF2IRD1*	0.29	4.19 × 10^−9^	0.01755	9.24 × 10^−2^	0.32	3.44 × 10^−11^	0	1
*TNN*	0.30	9.51 × 10^−10^	0.01615	9.29 × 10^−2^	0.27	7.20 × 10^−9^	0.03433	1.26 × 10^−3^
*FLT3*	0.30	8.37 × 10^−10^	0.00906	1.59 × 10^−1^	0.32	3.44 × 10^−11^	0	1
*CABP2*	0.32	4.12 × 10^−11^	0.00037	4.76 × 10^−1^	0.32	3.44 × 10^−11^	0	1
*ABTB1*	0.32	3.44 × 10^−11^	0	1	0.21	4.01 × 10^−11^	0.17969	1.90 × 10^−1^
*GAB2*	0.32	3.44 × 10^−11^	0	1	0.32	3.44 × 10^−11^	0	1
*GSN*	0.32	3.44 × 10^−11^	0	1	0.32	3.44 × 10^−11^	0	1
*KRTAP11-1*	0.32	3.44 × 10^−11^	0	1	0.32	3.44 × 10^−11^	0	1
*PSMD5*	0.32	3.44 × 10^−11^	0	1	0.30	9.46 × 10^−11^	0.00949	1.25 × 10^−1^

*geff*, Gene-specific effect estimate ($h_{geff}^{2}$); *geff_p*, significance of the gene-specific effect estimate; *h2r*, trait heritability estimate ($h_{r}^{2}$); *h2r_p*, significance of the trait heritability estimate.

**Table 2:** Top 10 most significant results for genes in a combined sample of 100 random and 12 causal genes

Rank	Strategy									
	
	Intragenic					Nonsyn				
	
	Gene	h2r	h2r_p	geff	geff_p	Gene	h2r	h2r_p	geff	geff_p
1	MAP4*	0.17	3.90 × 10^−6^	0.10955	7.20 × 10^−6^	MAP4*	0.18	7.00 × 10^−7^	0.10382	1.00 × 10^−7^
2	*OR9A4*	0.18	6.16 × 10^−11^	0.20337	4.64 × 10^−3^	TNN*	0.27	7.20 × 10^−9^	0.03433	1.26 × 10^−3^
3	LEPR*	0.26	4.16 × 10^−8^	0.04702	6.52 × 10^−3^	LSM12	0.15	3.40 × 10^−11^	0.26452	4.96 × 10^−3^
4	LRP8*	0.28	6.97 × 10^−9^	0.03575	6.55 × 10^−3^	NAT6	0.30	3.20 × 10^−11^	0.02515	1.16 × 10^−2^
5	*NAT6*	0.28	1.12 × 10^−10^	0.03592	8.39 × 10^−3^	AK123654	0.15	2.27 × 10^−10^	0.25783	1.37 × 10^−2^
6	*CCDC169-SOHLH2*	0.28	1.05 × 10^−8^	0.03547	2.25 × 10^−2^	OR2T27	0.28	1.05 × 10^−10^	0.04869	1.46 × 10^−2^
7	*OR2T27*	0.30	1.07 × 10^−10^	0.03072	4.20 × 10^−2^	HSPA9	0.15	8.69 × 10^−12^	0.26952	5.04 × 10^−2^
8	*CCDC169*	0.31	8.85 × 10^−10^	0.01913	4.53 × 10^−2^	LOC389493	0.21	1.52 × 10^−10^	0.16663	5.12 × 10^−2^
9	*GNG3*	0.18	1.85 × 10^−10^	0.20838	5.94 × 10^−2^	SRD5A1	0.32	2.25 × 10^−11^	0.01056	1.12 × 10^−1^
10	*GAS7*	0.28	1.91 × 10^−8^	0.03356	6.07 × 10^−2^	PSMD5*	0.30	9.46 × 10^−11^	0.00949	1.25 × 10^−1^

*geff*, Gene-specific effect estimate ($h_{geff}^{2}$); *geff_p*, significance of the gene-specific effect estimate; *h2r*, trait heritability estimate ($h_{r}^{2}$); *h2r_p*, significance of the trait heritability estimate.

*Known causal gene for SBP_1 in the simulated data set.

## Discussion

We performed variance component analyses using a novel approach to estimate the proportion of the trait phenotypic variance that can be attributed to a single gene. We first collapsed the genotypes from SNP variants into a GSRM that more closely approximates the correlations between related individuals at a gene-specific level. Figure 1 shows that there is substantial variation among genes in terms of the differences between TKE and gene-specific EKE values that had the potential to explain part of the trait variance. Thus, we then obtained gene-specific estimates of the $h_{geff}^{2}$ parameter and its significance from SOLAR, using the empirical GSRM.

Our results showed that the gene with the highest effect on the simulated SBP_1 trait was detected at a significance level that surpasses a conservative multiple testing threshold for the *p *values. Figure 2 shows that our test statistic was not inflated when evaluated under the null hypothesis using the Q1 trait and a random sample of genes. *MAP4 *was also consistently detected using the intragenic and nonsyn strategies (see Figures 1 and 2), with other causal genes ranking within our first top 10 results. This seems to suggest that our test is sensitive and specific enough for the detection of true-positive signals without enrichment of false-positive ones.

As a consequence of using a different strategy to select the SNPs for the estimation of the empirical GSRM, our results for *MAP4 *improved. *MAP4 *results were an order of magnitude less significant for the intragenic than for the nonsyn strategy. We believe that this is the result of rare functional alleles driving the EKE of the GSRM matrices for the nonsyn strategy without the noise introduced by shared noncoding alleles. In effect, the nonsyn GSRM matrices better approximate the gene's probability of identity-by-descent sharing, thus making our test a gene-specific empirical test of linkage that is also robust to the heterogeneity of the causal variants.

Finally, we want to note that our method is not restricted either to a particular measure of genetic identity or to its estimation on a gene-specific basis; identity-by-state and genomic regions, even if they are nonsyntenic [13], can potentially be used instead.

## Conclusions

We were able to obtain encouraging, proof-of-concept results from the application of our method to GAW18 data. We observed differences between the TKEs and their gene-specific empirical estimations. We obtained genome-wide significant results on the SBP_1 simulated trait for *MAP4 *that seem to indicate that our test is both specific and sensitive enough, and which also suggest that our method is behaving as a gene-specific empirical test of linkage.

## Competing interests

The authors declare that they have no competing interests.

## Authors' contributions

JB designed the overall study; JMP, MA, and JK conducted statistical analyses. JMP drafted the manuscript. All authors read and approved the final manuscript.
